# Impaired Antiviral and Type 3 Immune Responses in Crush Injury Patients With Earthquake‐Related Trauma

**DOI:** 10.1155/jimr/7908547

**Published:** 2026-08-26

**Authors:** Recep Civan Yuksel, Busra Seniz Demir, Mohammad Ahmad Houran, Mine Asan, Sahin Temel, Ahmet Safa Kaynar, Birkan Ulger, Tutkun Talih, Aliye Esmaoglu, Murat Sungur, Kürşat Gündoğan, Ahmet Eken

**Affiliations:** ^1^ Division of Intensive Care, Department of Internal Medicine, School of Medicine, Erciyes University, Kayseri, Türkiye, erciyes.edu.tr; ^2^ Erciyes University, Faculty of Medicine, Department of Medical Biology, Kayseri, Türkiye, erciyes.edu.tr; ^3^ Erciyes University, GENKOK Genome and Stem Cell Research Centre, Kayseri, Türkiye, erciyes.edu.tr; ^4^ Department of Anesthesiology and Reanimatio, Kayseri City Hospital, Ministry of Health, Kayseri, Türkiye, kayserisehir.saglik.gov.tr; ^5^ Department of General Surgery, School of Medicine, Erciyes University, Kayseri, Türkiye, erciyes.edu.tr; ^6^ Department of Anesthesiology and Reanimation, Division of Intensive Care, School of Medicine, Erciyes University, Kayseri, Türkiye, erciyes.edu.tr; ^7^ Department of Immunology and Microbiology, University of Colorado, Anschutz Medical Campus, Colorado, USA, colorado.edu

**Keywords:** alarmin, crush injury, DAMPS, earthquake, intensive care, interferon, type 3 immunity

## Abstract

**Background:**

Infections are frequent in critically ill patients with earthquake‐associated crush injury (CI) and contribute to poor outcomes, yet the immune pathways underlying posttrauma susceptibility remain poorly defined. We investigated longitudinal changes in damage‐associated molecular pattern (DAMP)/PAMP sensing and downstream inflammatory programs in CI patients requiring intensive care.

**Methods:**

Nineteen CI patients admitted to the ICU after the February 6, 2023 Kahramanmaraş earthquake and 18 age‐ and sex‐matched healthy controls were enrolled. Peripheral blood was collected on hospital days 7 and 21. DAMP/PAMP receptor expression was assessed by flow cytometry and RT‐qPCR, and pathway activation was evaluated by phospho‐flow (pSTING, pIRF3, pNF‐κB, and p‐cJun) in gated lymphocytes and monocytes. Plasma cytokines/alarmins and select DAMPs were quantified by multiplex enzyme‐linked immunosorbent assay (ELISA)/assays.

**Results:**

CI patients demonstrated broad remodeling of nucleic acid‐sensing programs, including reduced expression of TLR7, STING, and IRF7, with a discordant signaling pattern characterized by increased IRF3 phosphorylation, transiently reduced pSTING, and sustained reduction of p‐cJun, while NF‐κB activation remained comparatively stable. In plasma, IL‐33 and IFN‐α2 were reduced on Day 7, and type 3 immunity‐associated cytokines IL‐23 and IL‐17A were significantly decreased by Day 21, consistent with impaired antimicrobial barrier‐supporting responses. Circulating DAMPs were dynamically altered, with increased S100A9 and HMGB1 and decreased ATP following injury.

**Conclusions:**

Earthquake‐associated CI is associated with dynamic DAMP release and dysregulated innate sensing accompanied by suppression of antiviral and type 3 immune cytokine programs. These immune alterations may contribute to heightened vulnerability to secondary infections in critically ill CI patients.

## 1. Introduction

Crush syndrome, a life‐threatening complication of traumatic rhabdomyolysis, occurs when prolonged compression of skeletal muscle leads to tissue necrosis and systemic complications. It is classically defined as an ischemia‐reperfusion injury and is frequently observed in disaster scenarios, such as earthquakes, where individuals become trapped under debris [1]. Upon reperfusion, damaged muscle tissue releases cellular metabolites—including ATP, creatine kinase (CK), myoglobin, potassium, calcium, and nucleic acids—into the circulation, triggering systemic inflammation and multiple organ dysfunction [2].

Among critically ill patients with crush injury (CI), infections are common and significantly contribute to morbidity and mortality, especially when compounded by sepsis [3]. Several factors—open wounds, immune dysfunction, malnutrition, and invasive interventions—predispose patients to nosocomial infections [4, 5]. Sepsis in these settings is predominantly caused by multidrug‐resistant organisms, such as *Acinetobacter* spp. and *Pseudomonas aeruginosa*.

In the absence of infection, tissue damage itself can provoke sterile inflammation through the release of damage‐associated molecular patterns (DAMPs), which are sensed by pattern recognition receptors (PRRs) such as Toll‐like receptors (TLRs), RIG‐I‐like receptors (RLRs), and cytosolic DNA sensors including cGAS, STING, and AIM2 [6, 7]. These DAMPs include HMGB1, ATP, S100A9, nucleic acids, and heat shock proteins. Once recognized by PRRs or non‐PRR DAMP sensors, downstream signaling cascades activate transcription factors like NF‐κB and IRFs, leading to the production of proinflammatory cytokines, chemokines, and type I interferons [8–10].

Mitochondrial and nuclear DNA released from damaged cells can activate cytosolic sensors like cGAS‐STING and AIM2, which in turn induce type I IFNs and IL‐1 family cytokines through inflammasome activation [11, 12]. Similarly, cytosolic RNA sensors such as MDA5 and RIG‐I activate MAVS to initiate antiviral responses via IRF3/7 and TBK1 [13]. Cell‐surface receptors, including TLR4 and RAGE, also respond to extracellular DAMPs, propagating sterile inflammation [14].

In the context of crush syndrome, neutrophils rapidly infiltrate ischemic muscle following reperfusion, where they exacerbate injury through reactive oxygen species, proteases, and proinflammatory cytokines [15, 16]. Recent work has also highlighted the role of Th17 cells and their cytokines, particularly IL‐17A and IL‐23, in mediating inflammation and antimicrobial responses. These cytokines are critical in defending against fungi and extracellular bacteria and are influenced by upstream alarmins such as IL‐33 [17, 18].

Despite the known contribution of DAMPs to inflammation, the dynamics of DAMP receptor expression and associated signaling pathways in real‐life CI patients remain unexplored. No human studies to date have longitudinally evaluated how DAMP/PAMP sensors or their downstream immune mediators behave following earthquake‐associated trauma. Furthermore, while animal models suggest roles for TLR4, RAGE, and RIG‐I in sterile inflammation and organ damage following CI [19, 20], translational evidence in human subjects is lacking.

In this study, we aimed to investigate the expression and activation of DAMP and PAMP receptors, as well as associated cytokines and alarmins, in peripheral blood mononuclear cells (PBMCs) and plasma samples of critically ill patients with CIs resulting from the 2023 Kahramanmaraş earthquake. We performed longitudinal sampling at days 7 and 21 postinjury to elucidate dynamic immune changes that may underlie immune suppression, infection susceptibility, and adverse clinical outcomes in this population.

## 2. Materials and Methods

### 2.1. Ethics and Patient Information

This study was conducted in Erciyes University Hospital Intensive Care Units and the GENKOK Genome and Stem Cell Research Centre, Kayseri, Türkiye. The Ethics Committee of Erciyes University granted ethical approval for the study (Number 2023/679, date October 25, 2023).

In the period following the earthquake which occurred on February 6, 2023, 19 patients were identified who exhibited signs and symptoms indicative of a critical condition, including the need for intensive care and a diagnosis of CI. Blood and serum samples were collected from 18 patients on Day 7 after CI. Of these, six patients underwent repeat sampling on Day 21. An additional patient was sampled only on Day 21, resulting in a total of seven samples collected at the Day‐21 time point. This cohort formed the basis of the present study. Age‐ and sex‐matched 18 individuals were selected as the control group. Healthy controls were selected among age‐ and sex‐matched individuals without known acute infection, chronic inflammatory disease, autoimmune disease, malignancy, or immunomodulatory medication use at the time of blood collection.

All data were collected from the patients’ medical records and the hospital’s electronic medical system. Treatments received and clinical outcomes were recorded during the first 10 days of the patient’s stay in the ICU.

Demographic information such as age, sex, region where the earthquake occurred, and date of arrival at the ICU were recorded. The Glasgow Coma Score (GCS), APACHE II score, SOFA score, and modified NUTRIC score were also recorded.

The patients’ complete blood count and blood biochemistry values were recorded. These included creatinine (Cr), CK, myoglobin, C‐reactive protein (CRP), coagulation parameters, blood gas analysis, and urine analysis results.

### 2.2. PBMC Isolation

PBMCs were isolated using Lymphocyte Separation Medium (Cat# LSM‐A; 500 mL) following the manufacturer’s instructions. A total of 1 million cells were counted using the Trypan Blue exclusion method and preserved in TRIzol for subsequent RNA isolation. The rest of the isolated PBMCs were cryopreserved in 10% dimethyl sulfoxide (DMSO) (Cat# D2650). Additionally, plasma samples were collected for the enzyme‐linked immunosorbent assay (ELISA).

### 2.3. ELISA

Plasma samples that had been frozen at −80°C were retrieved and thawed at room temperature for the experiment. ELISA assays were performed using the Elabscience Human HMGB‐1 ELISA Kit (Cat# E‐EL‐H1554), ATP Chemiluminescence Assay Kit (Cat# E‐BC‐F002), and Human S100A9 ELISA Kit (Cat# E‐EL‐H6197) according to the manufacturer’s protocols. The levels of IFN‐α2, TSLP, IL‐1α, IL‐1β, GM‐CSF, IL‐11, IL‐12p40, IL‐12p70, IL‐15, IL‐18, IL‐23, IL‐27, and IL‐33 were measured using the LEGENDplex Human Cytokine Panel 2 (13‐plex) w/VbP V02 (Cat# 741378).

### 2.4. Real‐Time qPCR

One million cells preserved in TRIzol were retrieved from −80°C, and total RNA was extracted using the phenol‐chloroform method. Complementary DNA (cDNA) was synthesized from the RNA extracts using the iScript cDNA Synthesis Kit (Bio‐Rad, Cat# 1708891). Following cDNA synthesis, RT‐qPCR was performed using SYBR Green (Bio‐Rad, Cat# 1725124) on a LightCycler 480 Instrument (Roche) to detect PCR products. The relative gene expression of *TLR9*, *TLR7*, *TLR3*, *TLR4*, *MDA5* (*IFIH1*), *DDX58* (*RIG-I*), *cGAS*, *STING*, *IL-1*β, *IL-33*, *TNF-α*, *IFN-β1*, *IL-6*, *NF-κB*, *AIM2*, and *MAVS* was normalized to 18S ribosomal RNA and calculated using the ΔΔCT method. Primer names and sequences are listed in Table S1.

### 2.5. Phospho‐Flow Cytometry

PBMCs were counted after thawing and divided into six plates for six different experiments, three of which were phospho‐flow experiments. Cells were fixed with 4% paraformaldehyde (PFA) (100 µL/well) for 15 min at 37°C, followed by two washes with PBS. Permeabilization was performed using ice‐cold 100% methanol (150 µL/well) for 15 min on ice. Meanwhile, three antibody mixes were prepared. The first mix included Phospho‐STING (Ser366) (D8K6H) Rabbit mAb (Alexa Fluor 647), Phospho‐NF‐κB p65 (Ser536) (93H1) Rabbit mAb (Alexa Fluor 700), and purified anti‐Jun phospho (Ser73) antibody (Clone: A19012A/R/C). The second mix consisted of Phospho‐IRF‐3 (Ser396) (D6O1M) Rabbit mAb, PE anti‐AIM2 antibody (Clone: 3B10), and RIG‐I monoclonal antibody (Clone: 4G1B6). The third mix contained MDA5 recombinant rabbit monoclonal antibody (Clone: 33H12L34), FITC antihuman TLR7 antibody (Clone: S18024F), and PE‐Phospho‐IκB alpha (Ser32, Ser36) monoclonal antibody (Clone: RILYB3R). After permeabilization, the cells were washed twice with stain buffer. Fc blocking was performed using autologous human serum for 10 min at room temperature. Unconjugated antibodies were incubated with the cells for 30 min on ice in the dark, followed by a wash step. Secondary antibodies were then added according to the respective mixes and incubated for 30 min on ice in the dark. Finally, cells were washed, resuspended in 200 µL PBS, and analyzed using the FACSAria III flow cytometer.

### 2.6. Intracellular Cytokine Staining (ICS)

The PBMCs (1 million/well) were stimulated with phorbol myristate acetate (PMA), ionomycin, and Golgi Plug in 96‐well round bottom plates at 37°C, for 5 h in complete media (RPMI 1640 [Sigma]) supplemented with 10% fetal bovine serum (FBS) and antibiotics (Pen/Strep, Gibco) followed and processed for ICS. Using BD Cytofix/Cytoperm Plus intracellular staining kit (Cat# 554714), the cells were fixed and permeabilized. The first staining mix included cGAS (E5V3W) Rabbit mAb (Alexa Fluor 488) and TREM1 Polyclonal Antibody. The second mix contained FITC‐IFNβ Monoclonal Antibody (Clone: A1 [IFNb]) and Alexa Fluor 647 antihuman IL‐1β Antibody. The final mix comprised TRPM2 Antibody and RAGE Antibody. Unconjugated antibodies were processed, as described previously. Cells were analyzed on a FACSAria III, and data analysis was performed using FlowJo software.

### 2.7. Statistical Analysis

Flow cytometry data analyses were performed using FlowJo (10.0.7) software. Statistical analyses of FlowJo data and other datasets were conducted using GraphPad Prism 9 software. Data normality was checked via Shapiro–Wilk and Kolmogorov–Smirnov tests. If Gaussian distribution was achieved, we applied one‐way ANOVA for multiple comparisons with the Tukey multiple comparison correction method recommended by GraphPad Prism. In case of nonnormal distribution, nonparametric Kruskal–Wallis Test, with Dunn’s multiple comparison correction, was applied. Repeat sampling could not be performed for all patients because many were discharged or transferred to hospitals in different cities following the catastrophic earthquake. Since only six patients had paired Day 7 and Day 21 samples, the main figures were analyzed as cross‐sectional comparisons to allow the inclusion of all available Day 7 samples. Longitudinal paired analyses for the matched patients are presented separately in the Supporting Information Figures. The paired comparison between individuals sampled on both Day 7 and Day 21 was performed using a paired *t*‐test or Wilcoxon matched‐pairs signed‐rank test according to data distribution, after testing data normality with Shapiro–Wilk and Kolmogorov–Smirnov tests. In each case, using GraphPad Prism, outliers were calculated using the ROUT test (*Q* = 1) and excluded. The statistical significance values determined in GraphPad Prism 9 were assessed as follows:  ^∗^ *p* ≤ 0.05,  ^∗∗^ *p* ≤ 0.01,  ^∗∗∗^ *p* ≤ 0.001, and  ^∗∗∗∗^ *p* ≤ 0.0001.

## 3. Results

### 3.1. Patient Demographics and Clinical Information

The present study comprised 19 patients suffering from a critical CI. In addition, 18 age‐ and gender‐matched healthy subjects were included as a control group. The mean age of the patient and control groups was 39 ± 19 and 38 ± 19 years, respectively. Detailed patient demographic, laboratory, and clinical data (which include Acute Physiology and Chronic Evaluation Score, GCS, invasive mechanical ventilation, International Normalized Ratio, Bicarbonate, CRP, and CK levels) are shown in Table 1. The infectious outcomes and major ICU interventions for all CI patients, specifically, microbiologically documented infections, sites of isolation, causative pathogens, antibiotic exposure and duration, sepsis status, use of immunosuppressive medications, vasoactive drug administration, mechanical ventilation requirement, and mortality outcomes are given in Table 2.

**Table 1:** Demographic, clinical, and laboratory characteristics of the study participants.

Variables	Patients *N* = 19	Control *N* = 18
Age, SD (mean) (year)	39 ± 18	38 ± 18
Sex, *n* (%)		
Male	10 (53)	9 (50)
Female	9 (47)	9 (50)
APACHE II score, IQR (median)	12 (7–27)	—
GCS score, SD (mean)	13 ± 4	—
The type of oxygen support, *n* (%)		
Room air	2 (11)	—
Nasal mask	12 (63)	—
IMV	5 (26)	—
WBC (×1000) (0^3^/µL)	15.50 (12.00–23.74)	—
Hemoglobin (g/dL)	10.8 (8.9–13.0)	—
Platelets (×1000) (10^3^/µL)	199 (162–283)	—
BUN (mg/dL)	52.0 (24.5–66.0)	—
Creatinine (mg/dL)	1.22 (0.50–4.74)	—
CK (×1000) (µ/L)	17,711 (6757–76,611)	—
Myoglobin^a^ (×1000) (ng/mL)	3000 (883–15,060)	—
Sodium (mmol/L)	136 (128–145)	—
Potassium (mmol/L)	5.1 (4.1–6.7)	—
Chlorine (mmol/L)	103 (96–111)	—
LDH (U/L)	712 (552–2381)	—
D‐dimer (×1000) (µg/L)	6000 (3792–15,880)	—
Fibrinogen (×1000) (mg/dL)	406 (311–2480)	—
INR	1.14 (0.94–1.40)	—
HCO_3_ (mmol/L)	17.0 (13.3–21.0)	—
Lactate (mmol/L)	2.3 (1.2–4.0)	—
Urine pH	5.75 (5.00–6.00)	—
Blood pH	7.38 (7.24–7.44)	—
CRP (mg/dL)	129 (62–223)	—

Abbreviations: APACHE II, Acute Physiology and Chronic Health Evaluation Score; CK, creatine kinase; CRP, C‐reactive protein; GCS, Glasgow Coma Scale; HCO_3_, bicarbonate; IMV, invasive mechanical ventilation; INR, International Normalized Ratio.

^a^All laboratory values in the table are reported as the median (IQR) (25%–75%).

**Table 2:** Infection, treatment, and outcome information.

Patient	Culture result	Site of isolation	Culture‐isolated organism	Antibiotic use	Duration antibiotic therapy (days)	Indication of antibiotic therapy	Immunosuppressive medication	Vasoactive drug use	RRT	Intubation status	Mortality	Sepsis status	Sample days
1	Present	ETA + catheter	A + ^a^B	+	16	Treatment	Absent	Absent	Absent	Present	Absent	Present	7, 21
2	Present	ETA + catheter	^a^B + C	+	13	Treatment	Present	Present	CRRT	Present	Present	Present	7
3	Present	Catheter	^a^B	+	16	Treatment	Absent	Absent	CRRT	Absent	Absent	Present	7
4	Present	Wound site	^a^B	+	20	Treatment	Absent	Present	Absent	Absent	Absent	Present	7, 21
5	Absent	Absent	Absent	+	7	Empirical	Absent	Absent	Absent	Present	Absent	Absent	7
6	Present	Catheter	^a^B	+	12	Treatment	Absent	Absent	Absent	Absent	Absent	Absent	7
7	Absent	Absent	Absent	+	4	Empirical	Present	Present	IHD	Present	Present	Present	7
8	Present	Wound site	C	+	22	Treatment	Absent	Absent	Absent	Absent	Absent	Absent	7, 21
9	Present	Catheter	D	+	16	Treatment	Present	Present	Absent	Absent	Absent	Present	7, 21
10	Present	ETA	D	+	10	Treatment	Absent	Present	Absent	Present	Absent	Present	7
11	Absent	Absent	Absent	+	7	Empirical	Absent	Absent	Absent	Present	Absent	Absent	7
12	Present	Wound site	D	+	24	Treatment	Absent	Present	CRRT	Present	Present	Present	7
13	Absent	Absent	Absent	+	12	Empirical	Absent	Absent	Absent	Present	Absent	Absent	7
14	Present	ETA	D	+	14	Treatment	Present	Present	IHD	Present	Present	Present	7
15	Present	Wound site	E	+	16	Treatment	Absent	Present	Absent	Present	Absent	Present	7
16	Present	Catheter	^a^B + D	+	18	Treatment	Absent	Present	Absent	Present	Absent	Present	7, 21
17	Present	Catheter + wound site	^a^B + D	+	33	Treatment	Absent	Present	Absent	Absent	Absent	Present	7, 21
18	Absent	Absent	Absent	+	9	Empirical	Absent	Absent	Absent	Absent	Absent	Absent	7
19	Present	Catheter + wound site	A + D	+	120	Treatment	Present	Present	Absent	Absent	Absent	Present	21

*Note*: A: *Pseudomonas aeruginosa*, B: *Klebsiella pneumoniae*, C: *Stenotrophomonas maltophilia*, D*: Acinetobacter baumannii*, E: *Enterobacter cloacae*.

Abbreviations: CRRT, continuous renal replacement treatment; IHD, intermittent hemodialysis; RRT, renal replacement therapy.

^a^ESBL + the conditions.

Among the patients included in the study, the majority developed documented infections (73.6%) and/or sepsis during their ICU stay. Positive cultures were identified from endotracheal aspirates, catheter‐related samples, and wound sites, with multidrug‐resistant Gram‐negative organisms, particularly ESBL‐producing *Klebsiella pneumoniae* and *Acinetobacter baumannii*, being the predominant pathogens. Most patients required prolonged antibiotic treatment, and several required vasoactive support and mechanical ventilation. Only two patients (Pt2 and 12) received blood products; this was not added to Table 2. Collectively, these data support that CI patients are indeed prone to infections.

### 3.2. The Levels of DAMP Receptor Proteins and Gene Expression Were Altered After CI

Peripheral blood samples were collected from patients and controls on days 7 and 21 to investigate DAMP receptor pathways that could be activated by sterile inflammation in patients diagnosed with CI. Protein levels of DAMP receptor sensors were analyzed via flow cytometry by gating lymphocytes and monocytes separately in thawed PBMCs isolated from CI patients and healthy controls (Figure 1A and Figure S1A). Due to the unavailability of lineage‐specific markers (such as CD3 or CD14) for cost and laser source reasons, we gated lymphocytes and monocytes based on FSC and SSC plots instead of lineage‐specific marker expression, and throughout the text referred to them as morphologically identified lymphocytes/monocytes. Since Day 7 and Day 21 samples included six repeat samples, we also performed paired analyses for six patients, which are presented in Figure S2A. Although lymphocytes did not show a significant increase in the expression of TLR7 and MDA5 on Day 7 of CI, lymphocytes produced significantly more MDA5 on Day 21 in CI patients (*p* < 0.05) (Figure 1B, Figure S1B). In monocytes, TLR7 production decreased significantly (~two‐fold) on Day 7 of CI, whereas MDA5 showed a ~two‐fold increase on Day 21. Lymphocytes expressed high levels of RIG‐I, another key antiviral nucleic acid sensor, on both Day 7 and Day 21. In monocytes, RIG‐I expression increased significantly on both Day 7 and Day 21 of CI. TREM1 expression in monocytes also showed a significant increase, approximately doubling by Day 21.

Figure 1DAMP receptor protein and gene expression levels change after crush injury. (A) Histogram plots for DAMP receptor proteins of CI patients and controls: TLR7 (A1), MDA5 (A2), RIG‐I (A3), and TREM1 (A4). (B) Comparison of DAMP receptor proteins TLR7, MDA5, RIG‐I, and TREM1 in lymphocytes and monocytes between the control group and patients on days 7 and 21 after CI. Representative plots and quantified graphs are shown. (C) Quantification of nucleic acid sensors by relative gene expression in PBMCs of patients on days 7 and 21 after CI and controls. Comparison of *TLR3*, *TLR9*, and *IRF7* (C1); *STING*, *cGAS*, and *MDA5* (C2); *MAVS* (C3) nucleic acid sensors has been performed. Cohort size information: controls (CTRL) *n* = 18; Day 7 *n* = 18; Day 21 *n* = 6. Asterisks indicate *p* ^∗^ < 0.05,  ^∗∗^ < 0.01, ^∗∗∗^ < 0.001, and  ^∗∗∗∗^ < 0.0001.

**Figure:**
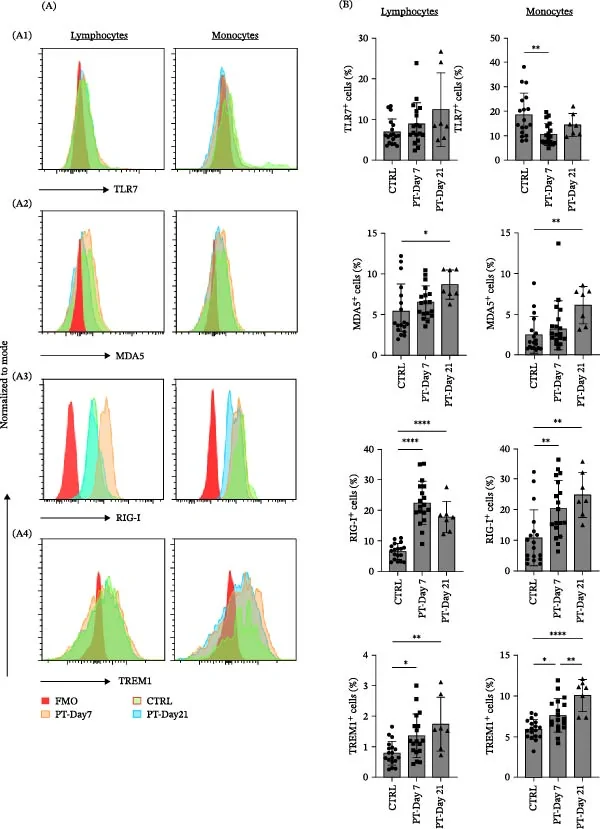

**Figure:**
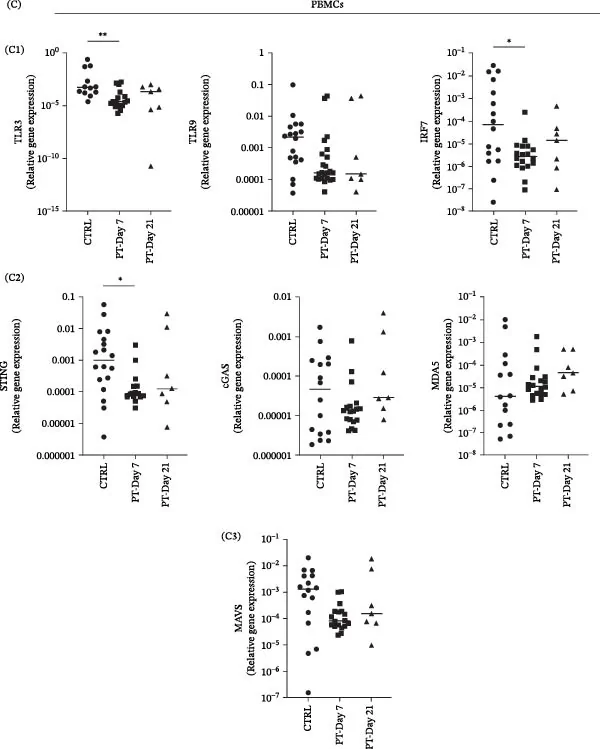

Gene expression analysis of nucleic acid DAMP/PAMP sensors in total PBMCs demonstrated overall suppression of several pathways important for antiviral responses and type I interferon induction (Figure 1C; primer details in Table S1), which persisted over 3 weeks of time (Figure S2B). The expression of TLR3 and TLR9, which recognize endosomal PAMPs, was reduced. IRF7, a key transcription factor downstream of TLR7/9 signaling, showed a significant decrease, particularly on Day 7. The cytosolic nucleic acid sensor STING and its upstream partner cGAS decreased on Day 7, with partial recovery by Day 21. In contrast, the MDA5 (IFIH1) gene expression showed a slight increase on days 7 and 21 in CI patients.

In summary, these data indicate that CI is associated with a mixed pattern of nucleic acid‐sensing dysregulation characterized by increased MDA5, RIG‐I, and TREM1 protein expression in monocytes, despite reduced expression of several interferon‐program components (TLR7, IRF7, STING, and cGAS) measured in bulk PBMCs. This apparent discordance may reflect cell‐type‐specific regulation, shifts in PBMC composition after severe trauma, and posttranscriptional mechanisms that differentially affect transcript abundance and protein expression.

### 3.3. Increased IRF3, Temporally Reduced STING, and a Sustained‐Reduced Jun Phosphorylation After CI

**Figure 2:**
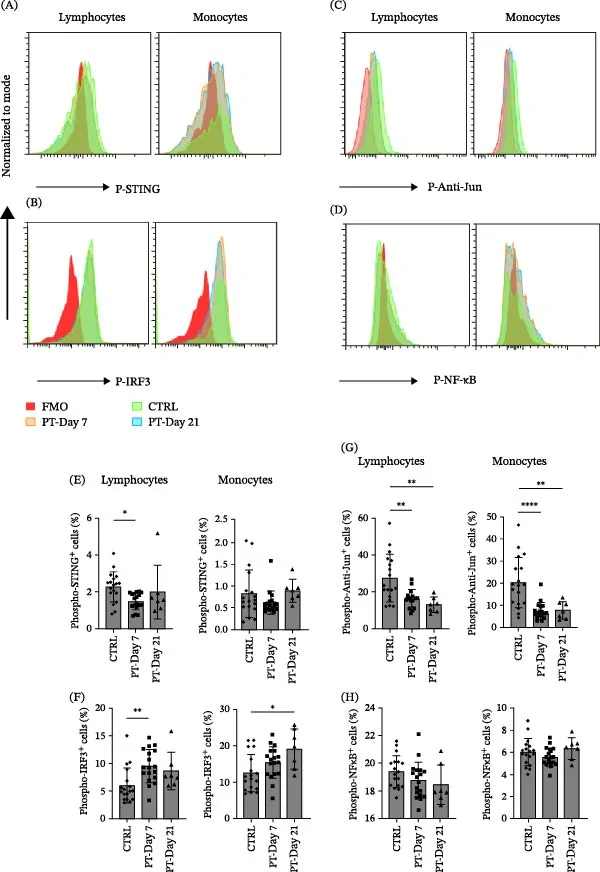
Increased IRF3, temporally reduced STING, and a sustained reduction Jun phosphorylation after crush injury. (A) Phosphorylated DAMP sensors were gated and shown as histogram plots from PBMCs of the controls and the patients after CI: phospho‐STING+, (B) phospho‐IRF3+, (C) phospho‐anti‐Jun+, and (D) NF‐κB+ cells. (E) Comparison of phosphorylated nucleic acid sensors according to lymphocyte and monocyte cells in patients on days 7 and 21 after CI and in controls in PBMCs: phospho‐STING + cells, (F) phospho‐IRF3+ cells, (G) phospho‐anti‐Jun+ cells, and (H) NF‐κB+ cells. Cohort size information: controls (CTRL) *n* = 18; Day 7 *n* = 18; Day 21 *n* = 6. Asterisks indicate *p* ^∗^ < 0.05,  ^∗∗^ < 0.01, ^∗∗∗^ < 0.001, and  ^∗∗∗∗^ < 0.0001.

To determine which inflammatory signaling pathways are activated or suppressed following CI, we assessed pathway activation states using phospho‐flow in all (Figure 2A–H and Figure S2A–H) and paired patient samples (Figure S3A–D). Activation of DAMP sensor pathways was evaluated separately in gated lymphocytes and monocytes. Phosphorylated STING levels were decreased in lymphocytes on Day 7 (Figure 2E) and partly returned to normal levels on Day 21 (Figure S3A), suggesting reduced STING pathway activation early after CI. In contrast, phosphorylated IRF3 showed a significant increase in lymphocytes on Day 7 and remained elevated in monocytes across time points (Figure 2F). Phospho‐cJun, a transcription factor associated with mitogenic signaling and immune cell activation programs, showed a consistent decrease across cell types after CI (Figure 2G). NF‐κB activation remained stable in all cell types at both time points (Figure 2H).

Together, these findings identify a distinctive signaling signature in CI, marked by heightened IRF3 phosphorylation despite early suppression of pSTING and sustained reduction in p‐cJun, suggesting the uncoupling of canonical nucleic acid‐sensing modules from downstream transcriptional programs involved in antiviral defense and immune‐cell activation.

### 3.4. Impaired Th17 Associated Cytokines and Alarmin IL‐33 Production After CI

**Figure 3:**
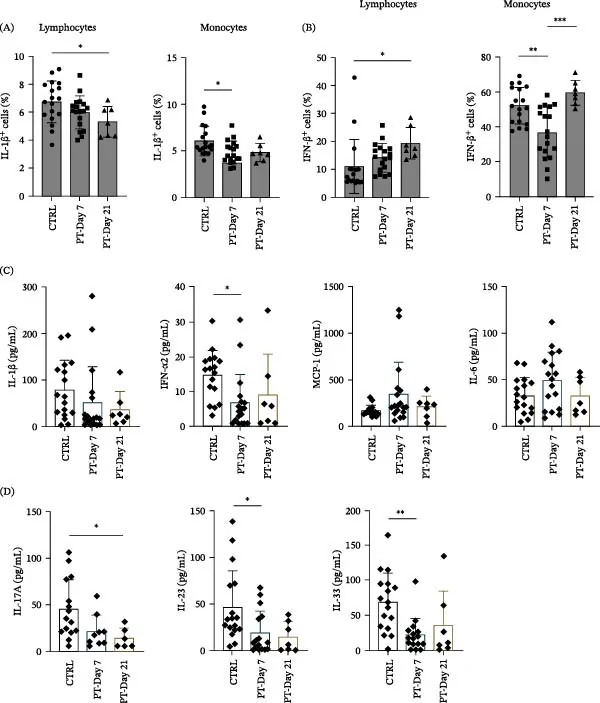
Impaired Th17‐associated cytokines and alarmin IL‐33 production after crush injury. (A) Comparative analysis of IL‐1β and IFN‐β in lymphocyte and monocyte cells in patients on days 7 and 21 after CI and in controls in PBMCs. (B) Percentage of IL‐1β+ and IFN‐β+ cells in lymphocytes and monocytes in patients on days 7 and 21 after CI and in controls in PBMCs. (C) IL‐1β, IFN‐α2, MCP‐1, and IL‐6, and (D) IL‐17A, IL‐23, and IL‐33 proinflammatory cytokine levels were measured from blood plasma with BioLegend Multiplex ELISA. Cohort size information: controls (CTRL) *n* = 18; Day 7 *n* = 18; Day 21 *n* = 6. Asterisks indicate  ^∗^ *p* < 0.05,  ^∗∗^ *p* < 0.01, and  ^∗∗∗^ *p* < 0.001.

ICS was performed on PBMCs to evaluate inflammatory cytokine responses following CI, and the data for all and paired CI patients on Day 7 and Day 21 are presented in (Figure 3A,B) and (Figure S4A–C), respectively. IL‐1β levels decreased progressively after CI, particularly in lymphocytes and monocytes (Figure 3A). In lymphocytes, IFNβ showed a progressive elevation after CI, while in monocytes, IFNβ decreased on Day 7 (Figure 3B andFigure S4B).

Plasma cytokines were quantified by multiplex ELISA from samples collected on days 7 and 21 (Figure 3C and Figure S4C). IL‐1β showed a slight downward trend over time, consistent with ICS. IFN‐α2, which is important for antiviral defense and T‐cell priming, was reduced on Day 7. MCP‐1 and IL‐6 showed a modest (nonsignificant) increase on Day 7, which went down on Day 21 (Figure S4C). Notably, the type 3 immunity‐associated cytokines IL‐17A and IL‐23 showed a progressive decrease after CI. IL‐33 levels were significantly decreased on Day 7, with levels trending toward control values by Day 21.

Overall, these data demonstrate that CI is followed by suppression of circulating cytokines that support antiviral responses (e.g., IFN‐α2) and type 3 immunity (IL‐23/IL‐17A), accompanied by an early drop in the epithelial alarmin IL‐33—all together consistent with the emergence of an immunosuppressed state that may increase susceptibility to secondary bacterial and fungal infections during ICU care.

### 3.5. Elevated DAMP Levels in the Peripheral Blood Plasma After CI

**Figure 4:**
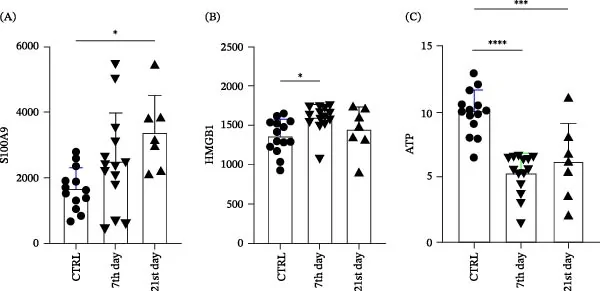
Increased S100A9 and HMGB1 DAMPs in the peripheral blood plasma after crush injury. (A) Comparison of S100A9, (B) HMGB, and (C) ATP levels between the control group and patients after log–log analysis in blood plasma collected on days 7 and 21 after CI. Cohort size information: controls (CTRL) *n* = 18; Day 7 *n* = 12–18; Day 21 *n* = 6. Asterisks indicate *p* ^∗^ < 0.05,  ^∗∗∗^ < 0.001, and  ^∗∗∗∗^ < 0.0001.

To quantify DAMP release following tissue and cellular damage in CI, we measured selected DAMPs in peripheral plasma again for all and paired patient samples (Figure 4A,C and Figure S5A–C). S100A9 levels increased progressively after CI (Figure 4A). HMGB levels increased significantly on Day 7 (Figure 4B). ATP levels decreased significantly in the plasma after CI (Figure 4C).

Collectively, these results confirm substantial systemic DAMP perturbation after CI, with increased S100A9 and HMGB1 alongside decreased extracellular ATP, supporting ongoing tissue damage and/or altered danger‐signal handling that may contribute to the immune remodeling observed in CI patients.

## 4. Discussion

Crush syndrome represents a severe form of ischemia–reperfusion injury characterized by extensive muscle necrosis, systemic release of intracellular contents, and subsequent multiorgan dysfunction. Historically, its pathophysiology has been defined by clinical observations from wartime and disaster‐related trauma, highlighting metabolic derangements, renal failure, and a high burden of infectious complications [1]. Subsequent clinical studies from large‐scale earthquakes, including the Marmara and Wenchuan earthquakes, established infections and sepsis as major contributors to mortality in CI patients, particularly in those requiring intensive care [3–5]. Indeed, our patient ICU data in the current report also confirm very high rates of infections and sepsis in CI patients. However, the molecular mechanisms underlying immune dysfunction and infection susceptibility in these patients have remained poorly defined.

In the present study, we provide the first longitudinal immunological profiling of earthquake‐associated CI patients, focusing on DAMP/PAMP sensing pathways and downstream cytokine responses. Our findings reveal a complex and dynamic immune landscape marked by profound dysregulation of nucleic acid sensing pathways, impaired antiviral responses, and suppression of type 3 immunity. These observations align with the broader concept that severe trauma disrupts immune homeostasis, resulting in a transition from early sterile inflammation to a prolonged immunosuppressive state [2, 21].

Tissue necrosis following a CI leads to the systemic release of DAMPs, including HMGB1, S100A9, ATP, mitochondrial DNA, and nuclear DNA, which act as endogenous danger signals [6, 7, 22]. These DAMPs are recognized by PRRs such as TLRs, RLRs, cGAS–STING, and inflammasome‐associated sensors, initiating inflammatory cascades that are essential for host defense but potentially pathogenic when dysregulated [8–10, 14]. Our observation of elevated circulating HMGB1 and S100A9 levels is consistent with animal models of CI demonstrating widespread DAMP release and systemic inflammatory signaling [19, 23, 24].

Despite this robust DAMP milieu, we observed a paradoxical suppression of key antiviral sensing pathways at the transcriptional level, including reduced expression of TLR7, IRF7, STING, and cGAS. A similar uncoupling between DAMP abundance and effective antiviral immunity has been described in trauma and aging, where chronic or excessive danger signaling leads to immune exhaustion and impaired interferon responses [25, 26]. In contrast, protein expression of RIG‐I and MDA5 was increased, particularly in monocytes, suggesting compensatory or cell‐type‐specific sensing of nucleic acid ligands. Prior studies have shown that RIG‐I can function as a DAMP sensor for myoglobin in CI‐associated acute kidney injury, activating NF‐κB and apoptotic pathways [20], supporting the relevance of this pathway in systemic injury responses.

Phospho‐flow analyses further revealed a distinctive signaling signature characterized by enhanced IRF3 phosphorylation despite transient suppression of phospho‐STING and sustained reduction of phospho‐cJun. IRF3 is a central transcription factor downstream of multiple nucleic acid sensors and can be activated independently of canonical STING signaling under cellular stress conditions [13]. Recent work has also highlighted mitochondrial and inflammasome‐associated routes to IRF3 activation, including STING–IRF3–NF‐κB crosstalk in sterile inflammatory settings [11]. Conversely, persistent suppression of cJun phosphorylation suggests impaired mitogenic and immune activation programs, consistent with trauma‐induced immune paralysis [27].

A particularly notable finding of our study is the progressive suppression of type 3 immune responses, evidenced by reduced circulating IL‐23 and IL‐17A levels. Th17 cells and related innate lymphoid populations are critical for protection against extracellular bacteria and fungi and play key roles in mucosal barrier integrity [17]. Experimental models of CI have demonstrated that modulation of the Th17/Treg axis influences renal injury and systemic inflammation, and therapeutic interventions that restore this balance can ameliorate organ damage [18]. Our data extend these observations to human CI patients and suggest that impaired type 3 immunity may contribute to the high incidence of secondary infections reported in clinical cohorts [3, 4].

The observed reduction in IL‐33 further supports this concept. IL‐33 acts as an epithelial alarmin that bridges tissue damage to immune activation and influences both type 2 and type 3 immune responses. Reduced IL‐33 levels may therefore exacerbate immune suppression by limiting the activation of protective cytokine networks following injury. Together with diminished IFN‐α2, these changes point to a broad suppression of antiviral, antibacterial, and antifungal immune programs in the postacute phase of CI.

Finally, our findings have potential therapeutic implications. Prior animal studies have demonstrated the benefits of targeting DAMP pathways, including neutralization of HMGB1 and RAGE blockade, in reducing organ damage after CI [19, 23]. Modulation of TREM‐1 signaling has also emerged as a promising strategy in sepsis and endothelial injury [28, 29]. In this context, restoring balanced nucleic acid sensing and type 3 immune responses rather than broadly suppressing inflammation may represent a rational approach to reducing infection risk and improving outcomes in CI patients.

Our study has some limitations. We lacked sampling during the hyperacute phase (0–48 h postinjury), which may have captured the peak of innate immune activation. Additionally, the sample size was limited to 19 patients, repeat sampling could not be applied to all patients due to patient discharge and patients being from different cities and the catastrophic nature of the earthquake due to the vastness of the affected region. Additionally, treatment‐related factors may have contributed to some of the observed immunological alterations and therefore represent a potential source of confounding. Lastly, comparison with critically ill noncrush trauma patients or ICU controls would have further strengthened the specificity of our findings.

## 5. Conclusions

In conclusion, this study provides the first longitudinal evidence that earthquake‐associated CI induces a sustained immunosuppressive state characterized by dysregulated DAMP sensing, impaired antiviral signaling, and suppression of type 3 immunity. These findings offer mechanistic insight into the high susceptibility to infection observed in CI patients and identify potential immunomodulatory targets for future therapeutic intervention.

## Author Contributions

Conceptualization, supervision: Murat Sungur, Kürşat Gündoğan, Ahmet Eken. Methodology: Busra Seniz Demir, Mohammad Ahmad Houran, Murat Sungur, Kürşat Gündoğan, Ahmet Eken. Software, validation, funding acquisition, project administration, resources: Kürşat Gündoğan, Ahmet Eken. Data curation: Recep Civan Yuksel, Busra Seniz Demir, Mohammad Ahmad Houran, Mine Asan, Sahin Temel, Aliye Esmaoglu, Ahmet Safa Kaynar, Birkan Ulger, Tutkun Talih, Kürşat Gündoğan, Ahmet Eken. Investigation: Recep Civan Yuksel, Busra Seniz Demir, Mohammad Ahmad Houran, Murat Sungur, Kürşat Gündoğan, Ahmet Eken. Formal analysis: Recep Civan Yuksel, Sahin Temel, Busra Seniz Demir, Mohammad Ahmad Houran, Aliye Esmaoglu, Ahmet Safa Kaynar, Tutkun Talih, Birkan Ulger, Murat Sungur, Kürşat Gündoğan, Ahmet Eken. Visualization: Busra Seniz Demir, Mohammad Ahmad Houran, Kürşat Gündoğan, Ahmet Eken. Writing – original draft: Recep Civan Yuksel, Busra Seniz Demir, Mohammad Ahmad Houran, Kürşat Gündoğan, Ahmet Eken. Writing – review and editing: Recep Civan Yuksel, Busra Seniz Demir, Mohammad Ahmad Houran, Mine Asan, Sahin Temel, Aliye Esmaoglu, Birkan Ulger, Ahmet Safa Kaynar, Tutkun Talih, Murat Sungur, Kürşat Gündoğan, Ahmet Eken.

## Funding

This study was supported by the Erciyes University Scientific Research Projects Unit (Grant TSA‐2024‐13353).

## Ethics Statement

This study was approved by Erciyes University Ethics Committee with the Approval Code 2023‐679.

## Consent

Written informed consent was obtained from patients or their legal guardians.

## Conflicts of Interest

The authors declare no conflicts of interest.

## Supporting Information

Additional supporting information can be found online in the Supporting Information section.

## Supporting information


**Supporting Information** Table S1: Primer sequences for relative gene expression. Figure S1: Gate strategy applied during flow cytometry analyses. Figure S2: Paired sample analyses for DAMP receptor protein and gene expression levels change after crush injury (related to Figure 1). (A) Comparison of DAMP receptor proteins TLR7, MDA5, RIG‐I, and TREM1 in lymphocytes and monocytes between the patients on days 7 and 21 after CI. Representative plots and quantified graphs are shown. (B) Quantification of nucleic acid sensors by relative gene expression in PBMCs of patients on days 7 and 21 after CI. Comparison of TLR3, TLR9, and IRF7 (upper); STING, cGAS, and MDA5 (middle); MAVS (bottom) nucleic acid sensors has been performed. Cohort size information: Day 7 *n* = 6; Day 21 *n* = 6. Asterisks indicate *p* ^∗^ < 0.05, ns: not significant. Outliers excluded via ROUT test (*Q* = 1) on GraphPad Prism. Figure S3: Paired sample analyses of phospho‐STING, JUN, IRF3, and NF‐κB after crush injury (related to Figure 2). (A) Percent phospho‐STING+ cells, (B) phospho‐IRF3+ cells, (C) phospho‐anti‐Jun+ cells, and (D) NFκB+ cells. Cohort size information: Day 7 *n* = 6; Day 21 *n* = 6. ns: not significant. Outliers excluded via ROUT test (*Q* = 1) on GraphPad Prism. Figure S4: Paired sample analyses of Th17‐associated cytokines and alarmin IL‐33 production after crush (related to Figure 3). Comparative analysis of IL‐1β and IFN‐β in lymphocyte and monocyte cells in patients on days 7 and 21 after CI among PBMCs bar graphs of IL‐1β (A) and IFN‐β (B) in lymphocyte and monocyte in patients on days 7 and 21 after CI among PBMCs. (C) IL‐1β and IFN‐β (top); MCP‐1 and IL‐6 (2nd row); IL‐17A and IL‐23 (3rd row); and IL‐33 (bottom) proinflammatory cytokine levels were measured from blood plasma with BioLegend Multiplex ELISA. Cohort size information: Day 7 *n* = 6; Day 21 *n* = 6. Asterisks indicate *p* ^∗^ < 0.05,  ^∗∗^ < 0.01. ns: not significant. Outliers excluded via ROUT test (*Q* = 1) on GraphPad Prism. Figure S5: Paired sample analyses of S100A9 and HMGB1 DAMPs in the peripheral blood plasma after crush injury (related to Figure 4). (A) Comparison of S100A9, (B) HMGB, and (C) ATP levels between the patients after log‐log analysis in blood plasma collected on days 7 and 21 after CI. Cohort size information: Day 7 *n* = 6; Day 21 *n* = 6. ns: not significant. Outliers excluded via ROUT test (*Q* = 1) on GraphPad Prism.

## Data Availability

The data that support the findings of this study are available from the corresponding author upon reasonable request.
