# Supplementary material for: Impaired Antiviral and Type 3 Immune Responses in Crush Injury Patients With Earthquake‐Related Trauma
Source: J Immunol Res. 2026 Aug 26;2026:7908547. doi: 10.1155/jimr/7908547 (PMC13519304; doi:10.1155/jimr/7908547)
Supplement: Supplementary file 1 [file JIMR-2026-7908547-s001.zip › Supplemental Table and Figure Legends.docx]

**Supplemental Table Legends:**

**Table S1:** Primer sequences for relative gene expression

**Supplemental Figure Legends:**

**Supplemental Figure 1.** Gate strategy applied during flow cytometry analyses

**Supplemental Figure 2.** Paired sample analyses for DAMP receptor protein and gene expression levels change after crush injury (related to Main Figure 1 **(A)** Comparison of DAMP receptor proteins TLR7, MDA5, RIG-1, and TREM1 in lymphocytes and monocytes between the patients on days 7 and 21 after CI. Representative plots and quantified graphs are shown. **(B)** Quantification of nucleic acid sensors by relative gene expression in PBMCs of patients on days 7 and 21 after CI, Comparison of *TLR3, TLR9, IRF7* (upper); *STING, cGAS, MDA5* (middle); *MAVS* (bottom) nucleic acid sensors has been performed. Cohort size information: Day 7 n=6; Day 21=6. Asterisks indicate p *<0.05, ns: not significant. Outliers excluded via ROUT test (Q=1) on Graph Pad Prism.

**Supplemental Figure 3.** Paired sample analyses of phospho-STING, JUN, IRF3 and NF-κB after crush injury (related to Main Figure 2). **(A)** Percentphospho-STING+ cells, **(B)** phospho-IRF3+ cells, **(C)** phospho-Anti-Jun+ cells, and **(D)** NFκB+ cells. Cohort size information: Day 7 n=6; Day 21=6. ns: not significant. Outliers excluded via ROUT test (Q=1) on Graph Pad Prism.

**Supplemental Figure 4.** Paired sample analyses of Th17 associated cytokines and alarmin IL-33 production after crush (related to Main Figure 3). Comparative analysis of IL-1β and IFN-β in lymphocyte and monocyte cells in patients on days 7 and 21 after CI among PBMCs bar graphs of IL-1β **(A)** and IFN-β **(B)** in lymphocyte and monocytes in patients on days 7 and 21 after CI among PBMCs. **(C)** IL-1β, IFN-β (top); MCP-1, IL6 (2^nd^ row); IL-17A, IL-23 (3^rd^ row); and IL-33 (bottom) proinflammatory cytokine levels were measured from blood plasma with BioLegend Multiplex ELISA. Cohort size information: Day 7 n=6; Day 21=6. Asterisks indicate p *<0.05, **<0.01.ns: not significant. Outliers excluded via ROUT test (Q=1) on Graph Pad Prism.

**Supplemental Figure 5.** Paired sample analyses of S100A9 and HMGB1 DAMPs in the peripheral blood plasma after crush injury (related to Main Figure 4) **(A)** Comparison of S100A9, **(B)** HMGB, and **(C)** ATP levels between the patients after log-log analysis in blood plasma collected on days 7 and 21 after CI. Cohort size information: Day 7 n=6; Day 21=6. ns: not significant. Outliers excluded via ROUT test (Q=1) on Graph Pad Prism.
