# Supplementary material for: Impaired Antiviral and Type 3 Immune Responses in Crush Injury Patients With Earthquake‐Related Trauma
Source: J Immunol Res. 2026 Aug 26;2026:7908547. doi: 10.1155/jimr/7908547 (PMC13519304; doi:10.1155/jimr/7908547)
Supplement: Supplementary file 1 [file JIMR-2026-7908547-s001.zip › Supplementary Table-1.docx]

| **Primer Name** | **5’>3’** |
| --- | --- |
| *TLR3* | ATGCACACAGCATCCCAAAG (FW)  AGGAAAGGCTAGCAGTCATCC (RV) |
| *TLR4* | CGAGGAAGAGAAGACACCAGT (FW)  CATCATCCTCACTGCTTCTGT (RV) |
| *TLR7* | AAACTCCTTGGGGCTAGATG (FW)  AGGGTGAGGTTCGTGGTGTT (RV) |
| *TLR9* | CGCCCTGCACCCGCTGTCTCT (FW)  CGGGGTGCTGCCATGGAGAAG (RV) |
| *RIG-1* | CACCTCAGTTGCTGATGAAGGC (FW)  GTCAGAAGGAAGCACTTGCTACC (RV) |
| *Cgas* | ATGTTTAAACTGGAAGTCCCC (FW)  AAGAAGTGTTACAGCAGGGCT (RV) |
| *STING* | GCCACCCCCTTGCAGACTTT (FW)  CCTGGTAGGCAATGAGGCGG (RV) |
| *IL-33* | GTGACGGTGTTGATGGTAAGAT (FW)  AGCTCCACAGAGTGTTCCTTG (RV) |
| *IL-1β* | GCCCATCCTCTGTGACTCAT (FW)  AGGCCACAGGTATTTTGTCG (RV) |
| *TNF-α* | GAAAGCATGATCCGGGACGTG (FW)  GATGGCAGAGAGGAGGTTGAC (RV) |
| *IFN-β1* | ATGACCAACAAGTGTCTCCTCC (FW)  GGAATCCAAGCAAGTTGTAGCTC (RV) |
| *IL-6* | GTCAGGGGTGGTTATTGCAT (FW)  AGTGAGGAACAAGCCAGAGC (RV) |
| *NF-κB* | GCAGCACTACTTCTTGACCACC (FW)  TCTGCTCCTGAGCATTGACGTC (RV) |
| *AIM2* | GCTGCACCAAAAGTCTCTCCTC (FW)  CTGCTTGCCTTCTTGGGTCTCA (RV) |
| *MAVS* | ATGGTGCTCACCAAGGTGTCTG (FW)  TCTCAGAGCTGCTGTCTAGCCA (RV) |
| *MDA5* | GCTGAAGTAGGAGTCAAAGCCC (FW)  CCACTGTGGTAGCGATAAGCAG (RV) |
| *18S* | GTAACCCGTTGAACCCCATT (FW)  CCATCCAATCGGTAGTAGGCG (RV) |

**Table S1**. Primer sequences for relative gene expression
